# Evaluating the efficacy of support groups in the metaverse for Ukrainian refugees: a protocol for a randomized clinical trial

**DOI:** 10.1186/s13063-024-08543-6

**Published:** 2024-10-19

**Authors:** Cezar Giosan, Cătălina-Maria Popoviciu, Saltanat Zhamaliyeva, Iuliana Zaborot, George Deac

**Affiliations:** 1 Department of Psychology and Cognitive Sciences, University of Bucharest, Panduri 90, Bucharest, Romania; 2 Sensiblu Foundation, Mogosoaia, Romania; 3 8Agora, Inc, Chardon, USA

## Abstract

**Background:**

The Ukrainian crisis, sparked by the Russian invasion, has generated one of the most extensive refugee crises in modern history. Addressing the mental health challenges of Ukrainian refugees is critical to promoting their resilience and successful integration into host communities. Traditional support group interventions might be challenging to implement for geographically dispersed populations, making the metaverse an innovative and inclusive platform for providing much-needed support to such populations.

**Methods/design:**

Displaced Ukrainian refugee adults (18 years or older) without current psychiatric diagnoses or current involvement in therapeutic interventions are included in the study. Participants are randomized to one of three conditions: (1) Metaverse Support Groups, (2) In-Person Support Groups, or (3) Waitlist. Both intervention groups (Metaverse and In-Person) undergo 5 support group sessions, and data are collected at baseline, mid-intervention, post-intervention, and 3-month follow-up. Primary outcomes are depressive symptomatology and anxiety. Secondary outcomes are perceived social support, well-being, and gender-based violence awareness.

**Discussion:**

To our knowledge, this is the first attempt to test the efficacy of support groups in the Metaverse for the Ukrainian refugee population. This study can thus add substantially to the body of knowledge on effective interventions and policies for refugees.

**Trial registration:**

ClinicalTrials.gov Identifier: NCT06142032 (https://clinicaltrials.gov/study/NCT06142032). Registered on November 8, 2023.

**Supplementary Information:**

The online version contains supplementary material available at 10.1186/s13063-024-08543-6.

## Background

The ongoing conflict in Ukraine has led to a significant number of displaced individuals seeking refuge in various countries, leading to one of the most pervasive conflict-based humanitarian crises in Europe since World War II. At the time of this writing, it is estimated that more than 6.2 million people have left Ukraine to seek refuge, from which more than 5.8 million are recorded in Europe [1]. Pre-migration and post-migration factors, such as trauma, loss, war violence, separation, lack of support networks, and psychosocial difficulties with adjustment in the host country, have been associated with increased susceptibility to mental health problems and poorer well-being for internally displaced Ukrainian refugees. Previous studies have shown associations with post-traumatic stress disorder, anxiety, depression, sleep disturbances, anger, and decreased quality of life [2–4]. Additionally, with restrictions imposed for the male population to leave the country, refugee communities are mostly comprised of women, with studies highlighting their increased vulnerability [3]. As previous ecological models have shown, ongoing stressors in refugees’ social ecology and displacement-related stressors have big effects on their mental health and well-being [5]. Addressing the mental health challenges of Ukrainian refugees is critical to promoting their resilience and successful integration into host communities.

Over the years, formally established support groups have proven their value for individuals’ well-being and mental health on the simple premise that people who share similar difficulties and stressors may understand one another better [6]. Support groups are an inexpensive and convenient, yet effective ways to receive support. Studies show a consistent pattern of the effectiveness of professionally-facilitated support groups for people struggling with mental illness [7]. Additionally, peer support groups as community-based interventions have been proven to be effective in addressing the psychosocial and mental health needs of refugees [8], with several studies showing improvements in their mental health, well-being, and psychosocial management [8].

However, traditional support groups for refugees come with some limitations: (1) interventions might be challenging to implement for geographically dispersed populations, (2) logistical constraints, (3) stigma associated with seeking support within a community or in-person setting may deter some individuals from participating or to meaningfully participate, (4) reduced flexibility in terms of accessibility, (5) cultural and linguistic limitations, and (6) lack of anonymity, which can inhibit open communication and deter individuals from seeking support due to fear of judgment or stigma [9–13].

In this context, increasing social support accessibility for Ukrainian refugees who struggle with mental health and psychosocial challenges is critical.

### Framework and rationale for the study

In recent years, online support groups have gained popularity, allowing for a more flexible and accessible way to facilitate mental health and psychosocial support. Several studies indicated that online chat support groups have a positive impact on mental health and well-being through empowerment [6], stigma—reduction [14], and peer support [6].

However, the vast majority of online support groups are un-moderated, very few of them are facilitated by a mental health specialist, or have a compelling raison d’être behind them, and very few of them have ever been tested in randomized clinical trials. Thus, it is largely unclear if they are as beneficial as traditional support groups or, perhaps, more importantly, if they might have unintended side effects (e.g., social avoidance). Additionally, to our knowledge, none has provided comprehensive evidence-based support for their efficacy on refugees. The technology format used for most support groups is either text-based (online group chats/forums on social media platforms) or through video conferencing platforms (e.g., Zoom). While these types of technological means may facilitate accessibility and flexibility, and manage geographical, cultural, and linguistic constraints, they may not provide full anonymity or may not have the potential to meaningfully re-create in-person environment and social cues.

There is a strong line of research showing the beneficial therapeutic effects of virtual reality therapy (VR therapy) [15]. Studies have shown that VR exposure therapy is efficient in treating post-traumatic stress disorder [15–17], phobias [15, 17, 18], substance-related disorders [15, 17], eating disorders [15, 17], psychosis [17], and even autism spectrum disorders [17]. In addition, evidence suggests that, compared to in-person interventions, VR-based group therapy can foster a sense of social presence and connection among participants, leading to an improvement in treatment outcomes [19].

More recently, the emergence of the metaverse has provided an exciting development for mental health support strategies [20]. While the evidence on the effectiveness of interventions delivered in the metaverse is still in its infancy, a few pilot studies have shown that applying metaverse-related technologies to deliver mental health programs for sexual dysfunctions, autism spectrum disorder, and eating disorders yields promising results [21–23].

### Objectives

The primary aim of this study is to evaluate the efficacy of Metaverse virtual support groups in improving the overall well-being of Ukrainian refugees. Secondary objectives include (1) understanding how virtual spaces impact Ukrainian refugees perceived social support and (2) whether such platforms can increase awareness of gender-based violence.

We aim to examine whether the eventual improvements observed in the active intervention groups are attributable to the interventions themselves rather than simply the passage of time. Therefore, we will compare the outcomes not only between the active intervention conditions but also with the waitlist control group, who are assessed similarly to the other groups but do not receive any intervention.

This study is critical in the current landscape for several reasons. Firstly, the metaverse offers a scalable way to reach a larger population of refugees who may be dispersed across various geographical locations, breaking the barriers of distance and time. Secondly, the anonymity provided by virtual environments may encourage more open dialogue about sensitive or stigmatizing issues, potentially leading to disclosures on sensitive topics and more effective emotional support and coping strategies. Lastly, utilizing immersive technology allows for a culturally and linguistically tailored approach, providing refugees not just with generic aid but with nuanced, community-specific support.

### Trial design

This protocol outlines a randomized clinical trial conducted to assess the efficacy of virtual support groups (i.e., groups meeting in the Metaverse) for Ukrainian refugees. The participants are randomly assigned to one of three conditions: (1) In-Person intervention group; (2) Metaverse intervention group; and (3) Waitlist. Stratification will consider factors such as age, gender, and trauma, to achieve balanced group distributions.

Major assessments are at baseline, mid-intervention, post-intervention, and 3-month follow-ups.

Our trial follows an equivalence framework and has a 1:1 allocation ratio, meaning participants are evenly distributed between the intervention and control groups.

## Methods

### Study setting

The design of this study complies with the Consolidated Standards of Reporting Trials (CONSORT) guidelines [24] and follows the Standard Protocol Items: Recommendations for Interventional Trials (SPIRIT) Statement 2013 [25] (see also Additional file 1 and Table 1).

**Table 1:**
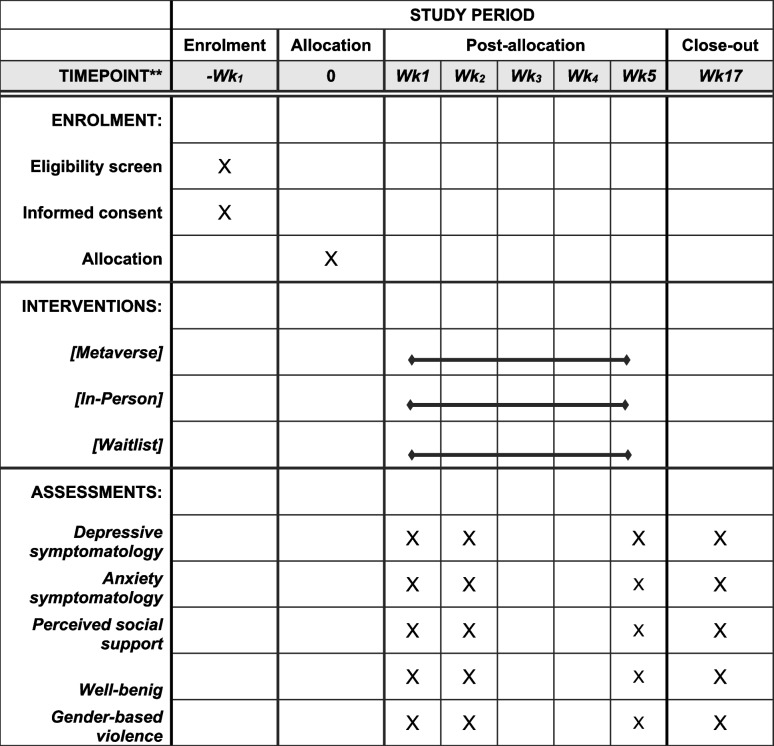
SPIRIT figure—schedule of enrollment, interventions, and assessments

The study population consists of Ukrainian refugees. The study is being implemented in collaboration with NGOs working with Ukrainian refugees.

### Eligibility criteria

Adult Ukrainian refugees (18 years or older) who are not formally diagnosed with a mental disorder, do not have scores suggestive of PTSD, and are not currently participating in any ongoing therapeutic intervention or mental health treatment were included.

### Interventions

Our research team designed the intervention protocol following evidence-based practices for support groups, specifically drawing from Cognitive Behavioral Therapy (CBT) principles [26]. Additionally, the protocol is based on guidelines from the United Nations High Commissioner for Refugees [27], Inter-Agency Standing Committee (IASC, 2007), the World Health Organization [28], and the Finish Refugee Council for support groups working with refugees [29]. Our intervention adopts a trauma-informed approach, acknowledging the unique and complex needs and experiences of individuals who have been exposed to traumatic events. The language used for the intervention sessions is Russian. This decision was made in consultation with previous Ukrainian refugees that the team worked with, as it is the preferred and most used language among this community. This language choice was deliberate to enhance inclusivity, allowing us to enroll a broader range of participants who could benefit from the intervention. To ensure agreement and comfort with the language choice, we included a question tapping into this, at the beginning of the screening process. We have observed no dropouts due to language concerns so far, supporting the argument for the effectiveness of our language inclusivity approach.

Both the Metaverse and In-Person conditions undergo a 1.5-h session each week for 5 weeks. Licensed psychotherapists facilitate both intervention conditions and follow the same protocol structure, which aims to improve the mental health and well-being of Ukrainian refugees and deliver psychoeducation on mental health and gender-based violence.

For both intervention conditions, the program has the following structure: (1) intake and the baseline assessments (described below) are conducted, (2) sessions 1 and 2, (3) mid-program assessment, (4) sessions 3, 4, and 5, (5) final assessment, (6) 3-month follow-up.

The general structure of a session is as follows: (1) *Introduction and Welcome*—a brief welcome and introduction to set the tone for the session, review of confidentiality and respectful communication guidelines, and group rules; (2) *Check-in*—participants share how they are feeling and briefly update the group on their current situation or experiences; (3) *Addressing the topic of discussion*; (4) *Sharing and support*—participants are encouraged to share experiences, thoughts, and feelings related to the topic of discussion, while group members provide supportive feedback, validation, and empathy to each other; (5) *Psychoeducation*—brief educational component related to the session’s topic, providing information, tools, or resources to enhance coping and resilience; (6) *Closure*—summary of the session, key takeaways, homework, reflections on the session, feedback, and next steps.

The Intake aims to assess participants if they are eligible for the study and introduce them to the goals of the program. The baseline assessment is conducted before the first session. A step-by-step guide about accessing and completing the survey is provided. Clarification of any questions or concerns are addressed. Session 1 focuses on facilitating an open discussion on participants’ needs, challenges, and cultural adjustment issues. It aims to create a safe and inclusive environment where participants can share their thoughts, concerns, and experiences, setting the tone for the support program. Session 2 focuses on the overall well-being of Ukrainian refugees and introduces strategies to enhance it. Session 3 focuses on perceived social support aiming at shedding light on its significance in our lives and providing practical tools to strengthen and enhance social networks. Session 4 focuses on mental health, with the aim of deepening participants’ understanding of mental health, reducing stigma surrounding mental health issues, and equipping them with essential knowledge and skills for promoting mental well-being. Session 5 is dedicated to raising awareness about gender-based violence (GBV), its various forms, and its profound impact on individuals and communities. Each session typically consists of 6 to 8 participants. The same 6–8 participants will attend each group session for the specific arm to which they are allocated. Each group will interact with the same members consistently from the first session to the last. Each arm is expected to have a minimum of two groups, and the formation of groups begins once a minimum of six participants have enrolled.

These groups convene weekly. To ensure that participants who miss a session are kept up to date, a briefing is conducted at the beginning of each session. This briefing provides a summary of the previous session’s discussions and activities, allowing all participants to stay informed and engaged, even if they were unable to attend the previous session.

### The Metaverse condition

For the Metaverse intervention condition, the Intake session provides an overview of the foundational principles guiding the utilization of the Metaverse as a platform for delivering support group sessions, and technical issues are addressed. Throughout the program, participants are prompted to share feedback during each session’s Check-in phase, highlighting any challenges or technical issues they may have encountered, which are promptly addressed.

### Generic description of the Metaverse condition

**Fig. 1:**
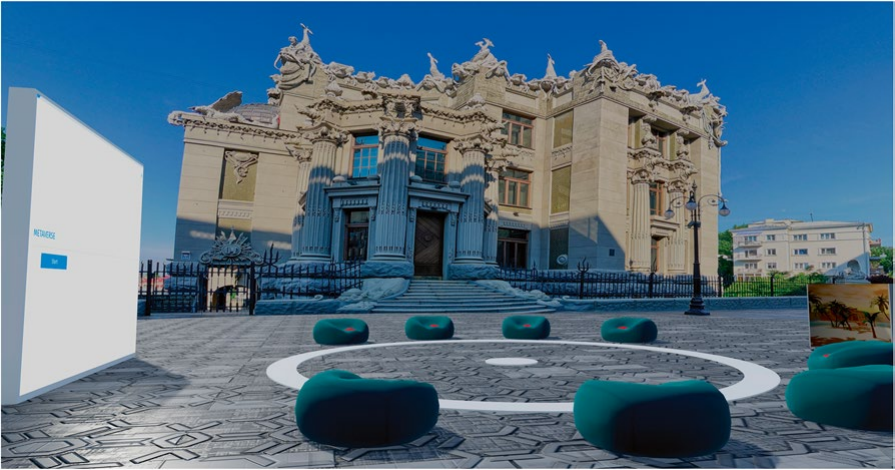
Metaverse meeting place (also see videoclip in the supplementary materials)

The participants in the Metaverse condition gather in a virtual city square in the city of Kyiv, close to Kyiv’s House with Chimaeras (see Fig. 1).

### Characteristics of the Metaverse

- Participants have the freedom to move around, take seats, and adjust their proximity to other attendees
- They can send private chat messages directly to the facilitator
- Participants have the option to interact with others by moving closer or further away
- They are free to make their seating arrangements and adjust as needed throughout the session

The Metaverse platform we utilize is accessed via a provided hyperlink, accessible through any browser on mobile or PC. Participants are directed to a registration/login interface upon clicking the link. Participants can leave sessions at any time, with their presence shown on the “online users” interface. Voice communication is proximity-based, with silent rooms available for private discussions. Access to the metaverse outside of sessions is controlled through a whitelist feature.

### The Waitlist

Participants in the Waitlist undergo the same set of assessments (minus those that tap into working alliance), under the same schedule as the active groups, after which time they will be given the possibility to join in-person group sessions.

### Outcomes and measures

#### Primary outcomes

The primary outcomes are the levels of depressive symptomatology and anxiety. We assess these outcomes by measuring changes in the total score from baseline to post-intervention. Specifically, we evaluate changes in the total scores of standardized assessment tools for depression and anxiety administered at baseline and post-intervention assessments.

#### Secondary outcomes

Well-being, gender-based violence awareness, and perceived social support constitute the secondary outcomes.

#### Measures

The instruments were translated into Russian, which is the most commonly used language within the Ukrainian refugee community in Romania. The translation process involved the standard back-translation method, and two native Russian speakers were engaged to ensure accuracy.

#### Screening measures

*The DSM-5-TR Level 1 Cross-Cutting Symptom Measure* is a self- or informant-rated measure that assesses important mental health domains across psychiatric diagnoses [30]. The adult version of the measure consists of 23 questions that assess 13 psychiatric domains, including depression, anger, mania, anxiety, somatic symptoms, suicidal ideation, psychosis, sleep problems, memory, repetitive thoughts and behaviors, dissociation, personality functioning, and substance use. Each item asks about how much (or how often) the individual has been bothered by the specific symptom during the past 2 weeks. Each item on the measure is rated on a 5-point scale (0 = none or not at all; 1 = slight or rare, less than a day or two; 2 = mild or several days; 3 = moderate or more than half the days; and 4 = severe or nearly every day).

*The experiences of the war and symptoms of trauma list of events* [31] is used to screen the war-related stressors. The scale has been validated in the study by Karatzias et al. [31]. The measure lists 34 war-related events (e.g., “I heard air raid sirens”), and participants are asked to indicate on a dichotomic—Yes (1) or No (0)—basis (scores range from 0 to 34) if they had experienced each event. As Karatzias et al. [31] instructed, participants are given the following instructions: “We wish to ask you about different things you may have experienced during the war. Below are descriptions of events that you may have experienced following the Russian attack on Ukraine on February 24th, 2022.” If exposed to multiple events, participants are asked to indicate the experience they found more distressing [31]. Higher scores reflect higher levels of war-related experiences.

#### Measures of primary outcomes

*The PHQ-9* [32] is used to assess the depressive symptomatology. The measure is a nine-question instrument designed to correspond to the *Diagnostic and Statistical Manual of Mental Disorder, Fourth Edition, Revised Text (DSM-IV-TR)* diagnostic criteria for major depressive disorder. Participants assign ratings on a scale of 0 to 3 based on how often they experienced specific items in the preceding 2-week timeframe (0—not at all; 3—nearly every day). The scores indicate the severity of depression, ranging from no depression to mild, moderate, moderately severe, or severe depression.

*The GAD-7* [33] is used to assess the severity of generalized anxiety disorder. The measure is a 7-question self-reported instrument designed to correspond to some of the *Diagnostic and Statistical Manual of Mental Disorder, Fifth Edition* diagnostic criteria for GAD. Participants assign ratings on a scale of 0 to 3 based on how often they experienced specific items in the preceding 2-week timeframe (0—not at all; 3—nearly every day). The scores indicate the severity of anxiety, ranging from minimal anxiety to mild, moderate, and severe.

#### Measures of secondary outcomes

*The Multidimensional Scale of Perceived Social Support (MSPSS; *[34]*)* is used to assess the Perceived Social Support. The questionnaire is a 12-item self-report instrument with items measured on a 7-point Likert scale (0—very strongly disagree; 7—very strongly agree). Participants are asked to indicate their preference for each statement provided in the measure. The statements refer to 3 subdomains—Significant Other, Family, and Friends (e.g., “There is a special person in my life who cares about my feelings.”)—and the scores can be calculated both for each subscale and as a total.

The *World Health Organization—Five Well-Being Index* (WHO-5; WHO, 1998) is used to assess the well-being of participants. The measure is a 5-item instrument consisting of statements that the respondents rate on a 6-point Likert scale (0—at no time; 5—all of the time). The statements are rated in relation to the past 2 weeks. The raw scores range from 0 to 25 and are multiplied by 100 to give the final score which ranges from 0 (the worst well-being) to 100 (the best well-being).

Gender-based violence awareness is assessed through 5 general knowledge questions about GBV. Participants will be asked to indicate the correct answer from a list of statements. Each correct answer receives 1 point. The total score ranges from 0 (no GBV awareness at all) to 5 (aware of GBV). The questions were developed in collaboration with an NGO focused on gender-based violence promotion among Ukrainian refugees.

#### Other measures

*Demographics* are collected through questions related to age, sex, gender, place of birth, country of displacement, assistance received while in the host country, marital status, and educational level. Questions regarding whether a family member died in the war, or the timeframe of arrival in the host country are also addressed. In the demographic section, questions concerning previous/actual involvement in therapeutic interventions and previous/actual mental disorders are also included.

The *Working Alliance Inventory – Short Form* (WAI-SR; [35]) was modified and is used to assess the therapeutic alliance within intervention groups. The measure is a 5-point Likert (1—Seldom; 5—Always) instrument consisting of 12 statements related to the relationships between client and therapist. For the present study, 6 items were adapted and are used to assess the alliance between support group facilitators and participants. These items pertain to the agreement on goals (e.g., “My support group leader and I agree on what is important for me to work on”), agreement on tasks (e.g., “My support group leader helps me to do what’s necessary to improve my situation”), and bond (e.g., “I feel comfortable working with my support group leader”), with each subdomain consisting of 2 questions.

#### Others

*The National Stressful Events Survey PTSD Short Scale* (NSESSS; APA, 2013) is a 9-item self-report on a 5-point Likert scale to assess post-traumatic stress disorder symptomatology.

### Participant timeline

**Fig. 2:**
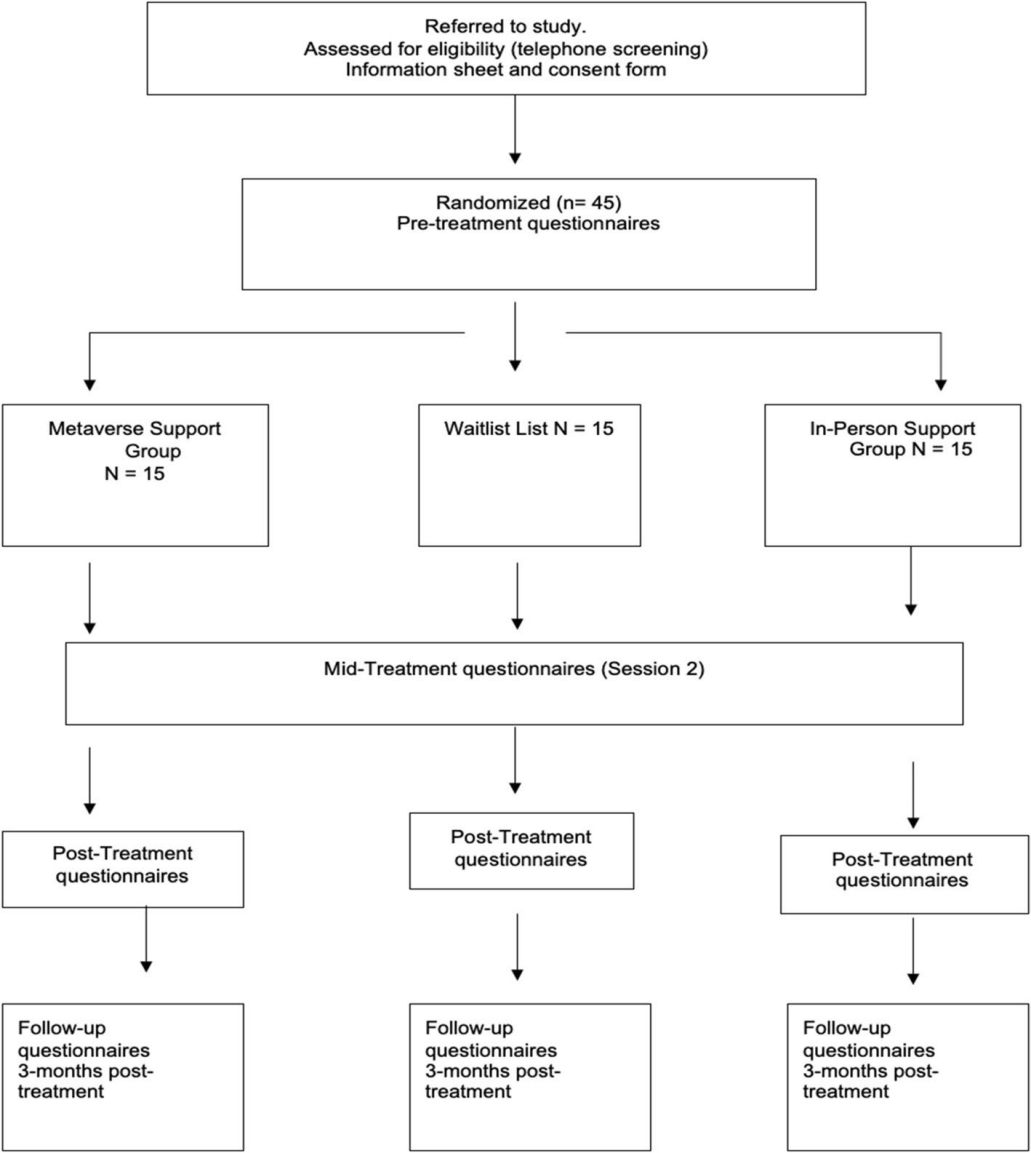
CONSORT flow diagram [36] showing subject allocation to the intervention conditions

Potential participants are assessed for eligibility through an initial assessment phase conducted by phone calls. If the inclusion criteria are met, written consent for participation in the study is obtained. After the initial assessment, the participants meeting the inclusion criteria are randomly assigned to one of the three conditions: (1) Metaverse Condition, (2) In-Person Condition, and (3) Waitlist condition (see flow diagram in Fig. 2). After the initial assessment, the subsequent measures consist of the instruments provided in the “Outcome measures” section. The participants are then randomized by a research assistant without being informed about their group allocation. A priori randomization is performed with a designated computer software (http://www.randomizer.org). Participants who do not meet the criteria for the study are referred to the appropriate entities and structures.

The study’s assessment and session timings are as follows: The baseline assessment session is delivered at enrollment, serving as the initial evaluation point. Between the baseline and the first session, approximately 1 to 2 weeks elapse. Subsequent sessions are held weekly thereafter. The mid-intervention assessment occurs after the second session but before the third, either on the day of the third session or 1 day prior. Similarly, the final assessment takes place after the fifth session, either on the same day or 1 day after. Additionally, a follow-up assessment is conducted 3 months after the conclusion of the intervention. In-person sessions are held at one of the NGO collaborators’ offices, providing a conducive environment for participant engagement and support.

#### Sample size

An a priori power analysis based on a medium effect size estimation [37, 38] indicated that a total of 45 participants are needed (planned main statistical test is ANOVA repeated measures, within-between interaction; *f* = 0.25; statistical power = 0.95; *α* error probability = 0.05; three groups, four main measurements (pre-intervention, mid-intervention, post-intervention, and follow-up). Power analysis was computed using the G*Power 3.1 program [39].

#### Recruitment

Prospective participants are recruited into the study by research assistants working with NGOs focused on Ukrainian refugees.

Collaborations with local NGOs, including the Sensiblu Foundation and regional Refugee Centers based in Bucharest, Romania, facilitate the recruitment process. These organizations provide direct access to the target population through their networks and community engagement efforts. An initial telephone discussion screens those people who are motivated to participate in the study. The screening interview focuses on the exclusion criteria (age, involvement in any current therapy process, and mental health diagnoses). Social media venues as well as posters and fliers are also used for the recruitment process. Recruitment strategies include community outreach, digital engagement, and collaboration with NGOs. Continuous efforts are maintained to meet the sample size requirement, with provisions for extended recruitment if necessary.

#### Assignment to interventions

The participants are randomly assigned to one of the three conditions (Metaverse, In-Person, Waitlist) using a sequence generated by the software randomizer.org.

The process of random allocation is executed by the project manager employing a straightforward random sequence, assigning one of three distinct numbers to each participant—either 1, 2, or 3, based on the number of the experimental condition.

Participants are enrolled by the project manager and trained facilitators from partner NGOs, with eligibility confirmed through thorough screening assessments. The project manager independently assigns participants to intervention groups, and allocation concealment is maintained by storing allocation lists as password-protected documents, accessible only after baseline assessments, ensuring that both participants and recruiters remain blind to group assignments until the intervention begins.

The Metaverse and In-Person groups are facilitated by two different psychotherapists, trained in evidence-based psychotherapies and group interventions, and who have at least 1 year of experience working with refugees. Both psychotherapists follow the same protocol structure (described in the “Interventions” section) to deliver the intervention.

The principal investigator and the statisticians conducting the data analysis remain unaware of the experimental condition until the study concludes.

#### Data collection, management, analysis

Eligible participants are assigned a unique identification number and asked to complete the assessment package (primary, secondary, and other outcomes, as well as demographics). The same set of measures are administered mid-intervention (after session 2), final intervention (after session 5), and 3 months after the final session (follow-up assessment).

To ensure high accuracy of data collection, all the measures are completed electronically, via an established survey solution such as QuestionPro, to minimize the risk of missing responses or errors in data entry. The facilitators are responsible for assisting the participants in this process, providing clarifications and explanations when needed.

To promote participant retention and follow-up completion, several strategies are being used. First, the participants receive a clear and complete description of the project, including, but not limited to, information regarding the goals of the support groups, their efficacy, and long-term effects based on evidence. Additionally, the participants will receive reminders about the upcoming session by email or other means to encourage the continuation of the program.

The improvement in the mental health and well-being scores within and between the groups will be examined using mixed-effects linear regression with a random intercept and slope over time (three assessments: baseline, after session 2, and post-treatment) and fixed effects for treatment assignment. The 3-month follow-up data will be analyzed using linear mixed effects model that incorporates random intercepts for participants and facilitators. Additionally, we may consider including random slopes for time if the data structure warrants it. If ICC analyses will indicate the presence of a hierarchical structure, we will proceed with conducting multilevel analyses. In addition, we will incorporate fixed effects for time and intervention condition and include any necessary covariates. We intend to perform longitudinal analyses in order to ascertain the long-term effects of the intervention. To evaluate changes in outcome measures from the initial measurement to each subsequent assessment, we plan to employ repeated measures ANOVA, where the data is complete, or mixed effects models, where data is incomplete. Moreover, we acknowledge that the linear mixed-effects model allows for the inclusion of covariates (we plan to control for demographics), and this flexibility makes it a robust alternative to repeated measures ANOVA, especially in complex datasets. The use of covariates was taken into account appropriately in the mixed-effects model, which is another reason we plan this approach when dealing with missing or incomplete data. The results of the other measures—demographic variables and therapeutic alliance as potential moderators and mediators—will be examined in an exploratory manner, utilizing regression models or mediation analyses. The error probability and the issue of multiple testing will be addressed either by employing the Bonferroni correction or the false discovery rate correction. Missing data—over 5%—will be handled with multiple imputation methods. Oversight and monitoring of the data collection process is conducted by the principal investigator and the project manager. Check-in regular meetings are held to review the progress of data collection, address any issues that may arise, and ensure compliance with the study protocol and ethical guidelines.

#### Monitoring study implementation

The management of unintended harm effects (i.e., a clinically significant increase in mental health symptomatology) and auditing is performed by the supervising clinical psychologist employed for this study, who monitors the clinical evaluations and support group sessions. If necessary, the supervisor can decide to terminate the intervention/clinical assessment and refer the participant to appropriate providers. Trained facilitators offer guidance and assistance to participants experiencing discomfort or adverse effects, ensuring their safety and well-being. Facilitators undergo comprehensive training before the intervention begins to maintain fidelity to the protocol. This training session is conducted by the principal investigator and the project manager designated for the intervention protocol. During this session, facilitators are provided with detailed instructions and guidelines on implementing the intervention protocol effectively. All facilitators adhere to this protocol at the beginning of each session. Protocol changes, if needed, will be communicated to the relevant parties by the PI.

Furthermore, to continually monitor and ensure adherence to the protocol, weekly check-in meetings are held between the project manager and the psychotherapists before each session. These meetings provide an opportunity to review session plans, address any questions or concerns, and reinforce adherence to the protocol. By providing initial training and ongoing support, we aim to equip facilitators with the knowledge and resources they need to deliver the intervention consistently and effectively, thereby enhancing the integrity and reliability of the study outcomes.

#### Ethics and dissemination

The study has been approved by the ethics committee of the University of Bucharest, Romania. Individuals expressing their interest in participating in the study access a link to a QuestionPro-type form, where they can find the informed consent as well as the instruments. The informed consent sheet includes information about the project coordinators, project purpose, and objectives, as well as information about the results and how they will be used, information about the participants role in the project, and what their rights are. It is specified that participation is entirely voluntary, and participants can exit the study at any time/stage without any consequences. Information about confidentiality and its preservation are also included as follows: all information received from participants will be treated as strictly confidential, in accordance with GDPR, the institution’s rules, and the ethical norms governing high-quality research worldwide; information recorded on paper or entered into the computer will be identified only by a code number; a list of participants’ names and codes will be kept separately and securely.

To enhance understanding and address any questions or concerns, our facilitators personally engage with each participant through phone calls or in-person meetings to explain the consent process in detail and take the consent from the participants.

Enrollment in the study takes place after participants agree, by checking boxes in the form, to the following aspects:

- That they have understood and received all necessary information to decide knowingly if they want to participate
- Voluntary agreement to enroll in the study
- Agreement regarding the use of data in the study: the understanding that the data will only be used for research purposes, in accordance with the informed consent form. Interested individuals can address further questions either to the project’s email address, the coordinator, or through the informed consent form

As with any study investigating mental health variables, there are ethical concerns that need to be addressed. First, the exclusion criteria exclude participants who are diagnosed with an actual mental disorder or pose an imminent danger to themselves or others. Also, potential participants who have scores indicative of posttraumatic stress disorder are also excluded. These people are referred to appropriate services and resources. Second, if a participant’s condition worsens during the intervention, the supervising clinical psychologist can opt to terminate the intervention ahead and make an appropriate referral. Third, since some of the support group topics might be sensitive and could generate delicate disclosures, facilitators are trained to manage this kind of situation and provide the needed support to participants.

#### Dissemination policy

The research team intends to publish the trial results in a peer-reviewed journal and also present the findings at conferences.

## Discussion

This protocol outlines a randomized clinical trial conducted to assess the efficacy of virtual support groups (i.e., groups meeting in the Metaverse) for Ukrainian refugees. The study aims to examine the effects of these virtual support groups on the mental health, social integration, and overall well-being of displaced Ukrainian refugees. Utilizing immersive technologies, this trial seeks to provide a safe, accessible, and culturally sensitive virtual environment for Ukrainian refugees to connect, share experiences, and receive support despite physical distance and geographical constraints.

To our knowledge, this is the first attempt to assess the efficacy of virtual support groups for Ukrainian refugees. The study can thus provide essential insights into the potential benefits of using the Metaverse in delivering support group sessions. This randomized trial will contribute to the development of targeted, scalable interventions and policies to promote the well-being of Ukrainian refugees.

There are, nonetheless, several limitations that must be acknowledged. First, as with many clinical trials testing psychological interventions, psychotherapist blinding is not possible. To minimize possible biases, the principal investigator and the project manager are not involved in any support group sessions. Second, the efficacy of virtual support groups may be influenced by the availability and accessibility of technology (e.g., familiarity with virtual platforms, access to a stable internet connection, etc.). To minimize the risk, participants receive technical assistance through the study and a short introduction on how to use the platform is in place. Third, virtual interventions might face higher rates of attrition and dropout compared to in-person. We monitor and report attrition rates and will conduct sensitivity analyses to assess the impact of dropouts on the results. Nevertheless, we acknowledge that, while Metaverse anonymity comes with several benefits, it can have some limitations for some people (e.g., participants not feeling comfortable not knowing who they are speaking to, maintaining confidentiality, whether the team can know for sure who is accessing the Metaverse platform). To minimize the risks, we have implemented several measures to ensure participant comfort and confidentiality within the Metaverse platform. Firstly, participants have the opportunity to meet the trainer before the sessions, either online or in-person, providing them with the opportunity to establish a rapport and familiarize themselves with the facilitator. Secondly, to ensure that the team can verify the identity of participants accessing the Metaverse platform, each participant is given a unique access code. This code is exclusively available to the participant and serves as a secure identifier when accessing the platform. By providing participants with individualized access codes, we can accurately track and monitor participation while maintaining confidentiality and ensuring that only registered participants have access to the sessions.

### Trial status

Preregistration: https://clinicaltrials.gov/study/NCT06142032; protocol version: 2, 15.02.24. Participant recruitment started on December 20, 2023. Recruitment end (estimated): April 2024. Randomization of the participants was performed on December 15, 2023.

### Study sponsor

Trial sponsor: University of Bucharest.

Contact name: Cezar Giosan, PhD (sponsor-investigator).

Address: Panduri 90, Bucharest, Romania.

Email: giosan@outlook.com.

Role of the study sponsor: supervision, design, implementation, execution, write-up for publication.

### Access to data statement

The principal investigator has direct access to the dataset, and the data dispersed to project team members will be blinded to any identifying information.

## Supplementary Information

Additional file 1.

Additional file 2.

## Data Availability

The datasets used and analyzed during the current study are available from the corresponding author on reasonable request.

## Abbreviations

GBV: Gender-based violence
PTSD: Post-traumatic stress disorder
GAD: Generalized anxiety disorder
DSM- 5: Diagnostic and Statistical Manual of Mental Disorders, Fifth Edition
PHQ: Patient Health Questionnaire
WHO: World Health Organization
NGO: Non-governmental organization
WAI-SR: Working Alliance Inventory Short Form
